# Synthesis of Ni-Co Hydroxide Nanosheets Constructed Hollow Cubes for Electrochemical Glucose Determination

**DOI:** 10.3390/s19132938

**Published:** 2019-07-03

**Authors:** Fengchao Sun, Shutao Wang, Yuqi Wang, Jingtong Zhang, Xinping Yu, Yan Zhou, Jun Zhang

**Affiliations:** 1 School of Materials Science and Engineering, China University of Petroleum (East China), Qingdao 266580, China; 2 School of Chemical Engineering, China University of Petroleum (East China), Qingdao 266580, China; 3 College of Science, China University of Petroleum (East China), Qingdao 266580, China

**Keywords:** Nonenzymatic, glucose sensor, electrochemical sensor, double hydroxides

## Abstract

Hierarchical Ni-Co double transition metal hydroxide nanosheets have been explored as an effective strategy for the design of nonenzymatic glucose sensors. Ni-Co hydroxide nanosheets constructed hollow cubes were successfully synthesized by using Cu_2_O cubes as templates and subsequently etched by Na_2_S_2_O_3_ to achieve a hollow cubic structure. The molar ratio between Ni and Co was tuned by varying the precursor ratio of NiCl_2_ and CoCl_2_. It was observed by transmission electron microscopy (TEM) that the increasing Ni precursor resulted in particle morphology, and the increasing ratio of the Co precursor resulted in more lamellar morphology. The sample with the composition of Ni_0.7_Co_0.3_(OH)_2_ displayed the best performance for glucose sensing with high selectivity (1541 μA mM^–1^ cm^–2^), low detection limit (3.42 µM with S/N = 3), and reasonable selectivity. Similar strategies could be applied for the design of other electrode materials with high efficiency for nonenzymatic glucose determination.

## 1. Introduction

Determination of glucose is of rising significance to the public because of its crucial function in human life activities [1,2,3]. Abnormal blood levels of glucose can cause serious health issues, including diabetes mellitus [4,5]. Therefore, fast and accurate determination of glucose is urgently demanded. The electroanalytical method has been proven to be one of the most promising ways for measuring the glucose concentration, with high sensitivity and relatively low cost, since Clark et al. introduced the concept of an enzymatic electrochemical glucose sensor more than 50 years ago [6]. Although three generations of enzymatic glucose sensors have been developed, the enzyme-based electrochemical sensor still suffers from low thermal stability, low chemical stability, high costs, and restricted working conditions (e.g., temperature and pH) due to the nature of the enzyme [7,8].

Therefore, enzyme-free electrochemical sensors have been developed and have demonstrated excellent sensory performances thanks to their thermal and chemical stability [9,10,11,12,13,14,15,16]. The mechanism of the nonenzymatic glucose sensor is similar to that of the enzymatic catalytic process, which oxidizes glucose to gluconolactone [17,18]. Therefore, multi-valence transition metal based electrocatalysts can be ideal for the electrochemical catalytic oxidation of glucose. Electrochemical oxidation of transition-metal-based compounds in the aqueous condition usually causes the formation of metal oxides or hydroxides on the surface of catalysts; therefore, the electrochemical catalytic oxidation of glucose on transition-metal-based compounds are usually achieved by the oxidation of surface hydroxides to oxyhydroxides [17]. More recently, double-metal hydroxides (DMHs) haven been intensively investigated for electrocatalytic purposes [19,20,21,22,23,24,25,26,27]. It was found that the introduction of the second metal ion into metal hydroxides can significantly tune the electronic structure, thus leading to faster electron transfer kinetics [28]. DMHs, including Ni/Al [27], NiCo [28], NiFe [29], CoFe [30], etc., have been studied for nonenzymatic glucose sensing. Additionally, the electrocatalytic performances of the electrode materials could also boost through the rational design of the hierarchical structures, owing to the increased active surface area, improved glucose diffusion pathways, and the tuned conductivity. Therefore, hierarchical hollow structures often show excellent glucose detection performances because of their high surface-to-volume ratios and large specific surface areas.

Herein, a hollow hierarchical structure is reported by arraying NiCo hydroxide nanosheets on Cu_2_O cubes followed by chemical etching. It was then used as an electrode material for a nonenzymatic electrochemical glucose sensor. Furthermore, the Ni and Co ratio was tuned by changing precursor ratios, and the composition of Ni_0.7_Co_0.3_(OH)_2_ displayed the best performance among other ratios towards electrochemical glucose oxidation.

## 2. Materials and Methods

### 2.1. Materials

In the experiment, all reagents were analytically pure and used directly. Cupric (II) chloride (CuCl_2_•2H_2_O), nickel (II) chloride (NiCl_2_•6H_2_O), cobaltous (II) chloride (CoCl_2_•6H_2_O), and sodium hydroxide (NaOH) were bought from Xilong Chemical Co., Ltd. (Guangdong, China). D-(+)-glucose (C_6_H_12_O_6_•4H_2_O), D-(+)-galactose (C_6_H_12_O_6_), L-ascorbic acid (C_6_H_8_O_6_), Polyvinylpyrrolidone K30 (PVP-K30), fructose, and ethanol absolute (C_2_H_6_O) were purchased from Sinopharm Chemical Reagent Co., Ltd. (Shanghai, China). Uric acid (C_5_H_4_N_4_O_3_) was bought from TCI (Shanghai, China). Acetaminophen was purchased from Shanghai Civi Chemical Technology Co., Ltd., and 5% Nafion solution was from Alfa Aesar (Shanghai, China).

### 2.2. Synthesis of Cu_2_O Cubes

The template of Cu_2_O cubes was synthesized as reported in a previous reference [31]. A total of 0.17 g CuCl_2_•2H_2_O was dissolved in 100 mL deionized water, then the solution was heated to 55 ℃ and stirred for 30 min. Afterward, 10 mL 2 M NaOH was added dropwise into the above solution to form a brown suspension. After stirring at 55 ℃ for 30 min, 10 mL 0.6 M ascorbic acid solution was added dropwise into the system and was continuously stirred at 55 ℃ for 3 h. The color of the suspension changed to brick red. The resulting precipitate was then collected by filtration, followed by washing with deionized water three times and ethanol once, and finally dried in a vacuum at 60 ℃ for 12 h.

### 2.3. Synthesis of Ni-Co Hydroxide Nanosheets

The hollow cubic Ni_x_Co_1-x_(OH)_2_ nanosheets were synthesized using a method reported previously [32]. Briefly, 10 mg Cu_2_O powder was dispersed with a solution containing 10 mL ethanol and 10 mL deionized water. After 30 min of ultrasonic treatment, 0.33 g PVP-K30 was added into the system. After another 30 min of ultrasonic treatment, 3.4 mg of CoCl_2_•6H_2_O and NiCl_2_•6H_2_O each was added into the suspension and stirred for 30 min. Then, 20 mL Na_2_S_2_O_3_ solution was dropped slowly into the system under stirring for 30 min. When the addition finished, the solution was stirred for 40 min and the color of the system changed from brick red to transparent green, proving the production of Ni_x_Co_1-x_(OH)_2_. The product was gathered by filtration. Afterward, it was washed with deionized water three times and ethanol once, and it was finally dried in a vacuum at 60 ℃ for 12 h. By changing the mass ratio of CoCl_2_•6H_2_O and NiCl_2_•6H_2_O, Ni_x_Co_1-x_(OH)_2_ could be produced with various designed compositions, such as Co(OH)_2_, Ni_0.3_Co_0.7_(OH)_2_, Ni_0.5_Co_0.5_(OH)_2_, Ni_0.7_Co_0.3_(OH)_2_, and Ni(OH)_2_.

### 2.4. Instruments

X-ray diffraction (XRD) was carried out with a Philips X’Pert diffractometer (PANalytical B.V., Almelo, Holland) under the conditions of Cu Kα radiation (λ = 0.15418 nm), 40 kV voltage, and 40 mA current. Transmission electron microscope (TEM) and high-resolution transmission electron microscope (HRTEM) analyses were tested on a JEM-2100UHR transmission microscope (JEOL, Tokyo, Japan) under a 200 kV testing voltage. X-ray photoelectron spectroscopy (XPS) was carried out using a VGESCALABMK II spectrometer (VG instruments, Suffolk, UK) with an Al Kα (1486.6 eV) photon source. Elemental mapping images were tested on an X-Max detector (Oxford instruments, Oxford, UK).

### 2.5. Electrochemical Tests

4 mg of Ni_x_Co_1-x_(OH)_2_ was dispersed in 900 mL of ethanol and 100 mL of Nafion. The suspension was sonicated for 10 min to gain a homogeneous ink. Carbon paper (CP) was produced with 6 M HCl solution, acetone, deionized water, and ethanol successively for 30 min under ultrasonic treatment. Then, 10 μL of the homogeneous ink was daubed on CP uniformly. After drying in air, the modified electrode was ready for electrochemical measurements. 

Electrochemical testing was carried out on a CHI 660E Electrochemical workstation (CH Instruments, Shanghai, China) with the standard three electrodes. A Pt electrode clip with Ni_x_Co_1-x_(OH)_2_/CP served as the working electrode. A mercury-mercuric oxide electrode acted as the reference electrode, and a Pt plate acted as the counter electrode. All electrochemical measurements were carried out in a 0.1 M NaOH electrolyte solution. 

## 3. Results and Discussion

### 3.1. Structural Characterization of Hierarchical Ni-Co Hydroxide Nanosheets

**Figure 1:**
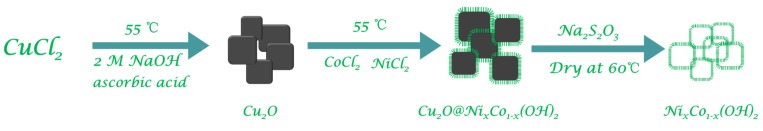
Schematic diagram of the synthetic procedure for the Co-Ni hydroxide nanosheets assembled the hollow cubic structures.

**Figure 2:**
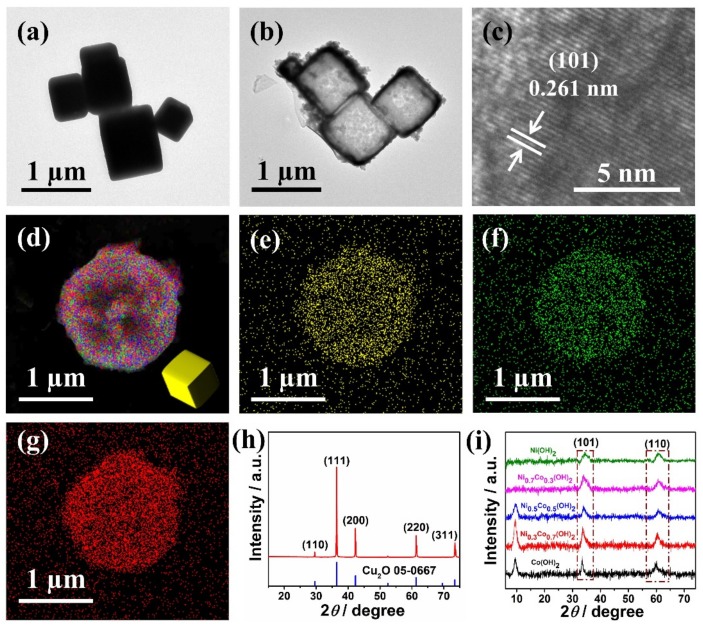
Transmission electron microscopy (TEM) images of (**a**) Cu_2_O and (**b**) Ni_0.7_Co_0.3_(OH)_2_ hollow cubes. (**c**) High-resolution transmission electron microscope (HRTEM) image of Ni_0.7_Co_0.3_(OH)_2_. (**d**–**g**) EDS mapping of Ni_0.7_Co_0.3_(OH)_2_ hollow cubes. The X-ray diffraction (XRD) patterns of (**h**) Cu_2_O and (**i**) Ni_0.7_Co_0.3_(OH)_2_ hollow cubes with different Ni-Co ratios.

The Co-Ni double-metal hydroxides nanosheets assembled hollow cube was synthesized by in situ growth of Co-Ni hydroxides on a Cu_2_O cube though a hydrothermal reaction, followed by Na_2_S_2_O_3_ etching to remove Cu_2_O (Figure 1). The ratios of Ni and Co were controlled by tuning the reactant ratio of NiCl_2_ and CoCl_2_. Figure 2a shows the typical TEM image of the cubic structure of Cu_2_O. The double-metal hydroxide of Ni_x_Co_1-x_(OH)_2_ was then directly grown on a Cu_2_O template with the removal of Cu_2_O to achieve a hollow cubic Ni_x_Co_1-x_(OH)_2_. The ratio between Ni and Co was controlled by the precursor ratio between Ni and Co. Figure 2b shows the hollow cubic Ni_0.7_Co_0.3_(OH)_2_. An increasing content of Co resulted in more distinct nanosheet morphology of Ni_x_Co_1-x_(OH)_2_ (Figure S1). The HRTEM image (Figure 2c) shows a clear lattice fringe with 0.261 nm d-spacing, which consisted of the (101) crystal plane of Ni(OH)_2_. Energy dispersive spectrometer (EDS) based elemental mapping (Figure 2d–g) showed evenly distributed Ni, Co, and O, indicating that the Ni-Co hydroxide was successfully synthesized. Figure 2h shows the XRD patterns of Cu_2_O cubic templates, which consisted of the reference data (PDF No. 05-0667). Figure 1i shows the XRD patterns of Ni-Co hydroxides with different Ni-Co ratios. The peaks of (101) and (110) crystal planes were clearly observed for Co(OH)_2_, Ni(OH)_2_ and Ni-Co hydroxides, with slight shifts in the diffraction angles, suggesting continuous variations of Ni-Co ratios. It is clear that the increasing content of Co in Ni-Co hydroxide results in the appearance of a peak at 9.5°, suggesting a nanosheet morphology predominates when Co content is high. This agreed well with the TEM findings.

**Figure 3:**
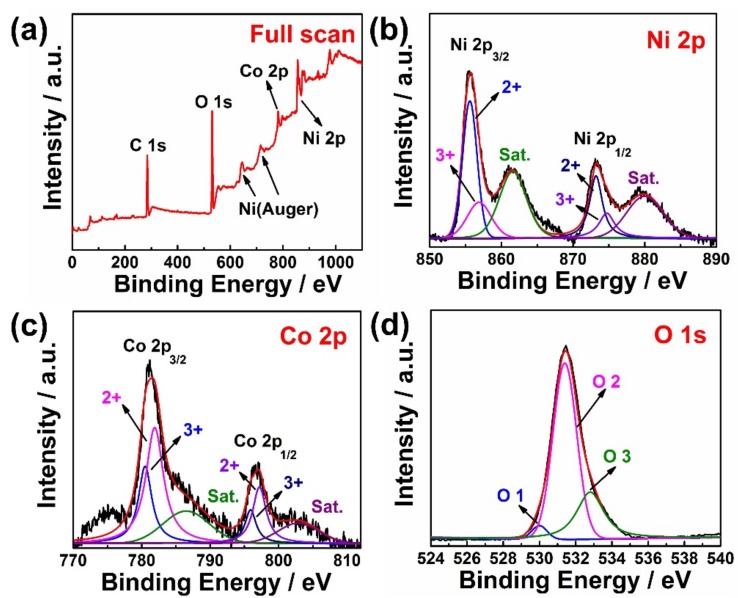
X-ray photoelectron spectroscopy (XPS) spectra of Ni_0.7_Co_0.3_(OH)_2_ (**a**) full survey and in the core spectrum region of (**b**) Ni 2p, (**c**) Co 2p, and (**d**) O 1s.

X-ray photoelectron spectroscopy (XPS) analyses were carried out to further verify the surface composition and oxidation states of Ni_0.7_Co_0.3_(OH)_2_ hollow cubes. Figure 3a shows the complete XPS spectrum survey of Ni_0.7_Co_0.3_(OH)_2_, where there are featured peaks of Ni 2p, Co 2p, O 1s and C 1s. The core-level spectrums for Ni 2p, Co 2p, and O 1s were further analyzed to acquire detailed oxidation states and local electronic environments for Ni, Co, and O. Figure 3b displays the core-level spectrum in the Ni 2p region. Six curves were deconvoluted in order to obtain a reasonable fitting for Ni 2p. The peaks at 855.6 and 873.2 eV can be ascribed to the Ni^2+^ oxidation state, and another two peaks with the binding energies of 856.9 and 874.7 eV can be assigned to the Ni^3+^ 2p spin orbits. The core level XPS spectrum of Co 2p is shown in Figure 3c. Two valence states of Co^3+^ and Co^2+^ for cobalt species were found. The peaks at 780.5 and 795.9 eV are ascribed to the 2p_3/2_ and 2p_1/2_ states of Co^3+^. Similarly, the peaks appearing at 781.9 and 797.2 eV are attributed to the 2p_3/2_ and 2p_1/2_ of Co^2+^ pieces. These results confirm that both Co and Ni elements have mixed oxidation states in Ni_0.7_Co_0.3_(OH)_2_ samples, which agrees well with previous reports [33]. This composition is similar to that of Ni-Co double-layered hydroxide [28]. Furthermore, the XPS core-level spectrum of O 1s shown in Figure 3d displays three deconvoluted peaks with binding energies of 530.3, 531.4, and 532.8 eV, which can be attributed to the metal-bonding oxygen, hydroxyl oxygen, and surface oxygen caused by physical or chemical adsorption, respectively [34].

### 3.2. Electrochemical Characterization of Hierarchical Ni-Co Hydroxide Nanosheets

**Figure 4:**
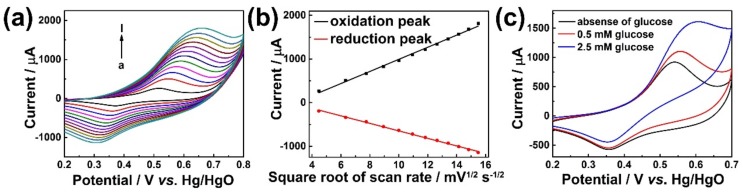
(**a**) Cyclic voltammograms of the Ni_0.7_Co_0.3_(OH)_2_/carbon paper (CP) electrode scanned from +0.2 to +0.8 V vs. Hg/HgO in 0.1 M NaOH at variable scan rates. (**b**) The corresponding Randles–Sevick plot to (**a**). (**c**) The CV curve of the Ni_0.7_Co_0.3_(OH)_2_ drop-casted carbon paper electrode in absence and presence of 0.5 and 2.5 mM glucose.

Electrochemical methods were further carried out on the hierarchical Ni-Co hydroxide nanosheets in order to understand their redox behaviors. Figure 3a shows the cyclic voltammograms of the Ni_0.7_Co_0.3_(OH)_2_ drop-casted carbon paper (Ni_0.7_Co_0.3_(OH)_2_/CP) electrode scanned from +0.2 V to +0.8 V vs. Hg/HgO in 0.1 M NaOH at variable scan rates. A pair of oxidation and reduction peaks can be observed at +0.52 V and +0.37 V vs. Hg/HgO, respectively. Since Ni predominated in this compound and it is mainly consisted of the +2 oxidation state, the oxidation peak at +0.52 V vs. Hg/HgO was mainly attributed to the electrochemical oxidation of Ni^2+^. The cathodic peak at +0.37 V vs. Hg/HgO can be assigned to the reduction of surface-oxidized Ni^3+^. Since the oxidation of Co^2+^ occurs at similar electrode potential compared to Ni^2+^, the presence of Co^2+^ in the catalyst can enhance the oxidative current density of Ni_0.7_Co_0.3_(OH)_2_, thus leading to enhanced sensitivity towards glucose sensing. Furthermore, the slight shifts in peak potentials with increasing scan rates suggest that the electrode process is rather sluggish. This is reasonable for most of the transition metal oxides and hydroxide materials since they usually suffer from low intrinsic conductivity. Figure 4b shows the corresponding Randles–Sevick plot of Figure 4a. It shows a clear linear dependence of peak currents on the square root of scan rates, suggesting a diffusion-controlled redox process. This is reasonable because the electrochemical oxidation of Ni_0.7_Co_0.3_(OH)_2_ involves hydroxide ions, which need to diffuse from the bulk solution to the surface of the electrode material. Figure 4c shows the CV curves of the Ni_0.7_Co_0.3_(OH)_2_/CP electrode in the absence and presence of glucose. It is clear that in the presence of 0.5 and 2.5 mM of glucose, the oxidation peak current increased significantly. The increase of peak current upon the addition of glucose suggests an electrochemical catalytic (EC’) electrode process. Thus, the electrode reaction can be summarized as follows [9,35]:

(1) $${\mathrm{Ni}}_{0.7}{\mathrm{Co}}_{0.3}{\left(\mathrm{OH}\right)}_{2}+{\mathrm{OH}}^{-}\to {\mathrm{Ni}}_{0.7}{\mathrm{Co}}_{0.3}\mathrm{OOH}+e^{-}+H_{2}O;$$

(2) $${\mathrm{Ni}}_{0.7}{\mathrm{Co}}_{0.3}\mathrm{OOH}+\mathrm{glucose}⇌{\mathrm{Ni}}_{0.7}{\mathrm{Co}}_{0.3}{\left(\mathrm{OH}\right)}_{2}+\mathrm{glucolactone};$$

where (1) represents the electrochemical oxidation of Ni_0.7_Co_0.3_(OH)_2_ (E), and (2) represents the heterogeneous catalytic process occurring on the surface of the electrode (C’).

**Figure 5:**
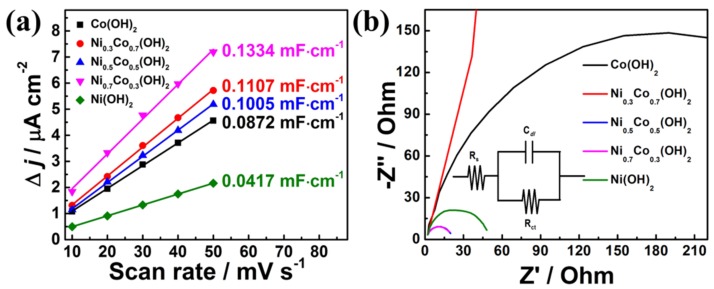
(**a**) The double-layer capacitance (*C_dl_*) of different Ni_x_Co_1-x_(OH)_2_/CP electrodes, which were obtained from the cyclic voltammetry scanned in the non-Faradaic region. (**b**) Nyquist plots of Ni_x_Co_1-x_(OH)_2_/CP in 0.1 M NaOH at their respective open circuit voltages.

Although it is rather difficult to determine the exact active surface areas, it is still reasonable to reveal the total surface areas that are exposed in the electrolyte. The different Ni_x_Co_1-x_(OH)_2_/CP electrodes were firstly evaluated using CV by scanning in the non-Faradaic potential range (Figure S2). The corresponding plot of current difference against scan rates is shown in Figure 5a. It is clear that Ni_0.7_Co_0.3_(OH)_2_ exhibited the highest *C_dl_* value, suggesting the largest surface areas exposed in the electrolyte solution. The large surface area can thus create more redox centers at the material surface, which can lead to increased current density. Thus, the increased current density may result in enhanced sensory sensitivity. In order to reveal the insight of electrode kinetics, electrochemical impedance spectroscopy (EIS) analyses were carried out for Ni_x_Co_1-x_(OH)_2_/CP. The Nyquist plots of different electrodes are shown in Figure 5b, and the corresponding equivalent circuit is shown in the insertion. The *Rs* values differed only slightly for the series resistance of all five samples, indicating that the electronic conductivity of the different Ni_x_Co_1-x_(OH)_2_/CP are nearly the same. Moreover, the charge-transfer resistance (*R_ct_*) of Ni_0.7_Co_0.3_(OH)_2_ was equal to 21.27 Ω, which is much smaller than other electrode materials, suggesting much faster charge transfer kinetics of Ni_0.7_Co_0.3_(OH)_2_ at the interface between the electrocatalyst and electrolyte compared to other samples. Therefore, Ni_0.7_Co_0.3_(OH)_2_ exhibited the largest electrochemical surface area and the fastest electrode kinetics among other materials; thus, it can be an excellent candidate for electrocatalytic glucose oxidation.

### 3.3. Sensory Evaluation of Ni_x_Co_1-x_(OH)_2_/Carbon Paper (CP) Towards Glucose Detection

**Figure 6:**
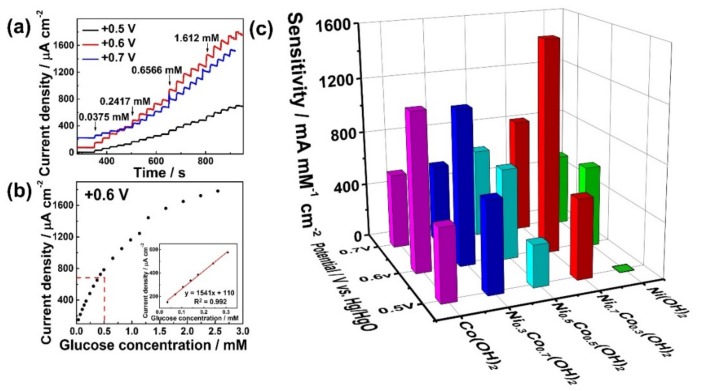
(**a**) Current responses of Ni_0.7_Co_0.3_(OH)_2_/CP at +0.5, +0.6, and +0.7 V vs. Hg/HgO with the changing concentration of glucose. (**b**) The calibration plot of glucose concentration to the current density of (**a**); the illustration shows the linear section. (**c**) The sensitivities of different electrodes obtained by the slope of the linear part of different samples at +0.5, +0.6, and +0.7 V vs. Hg/HgO.

Ni_0.7_Co_0.3_(OH)_2_/CP was firstly evaluated for glucose detection using chronoamperometry with stirring in 0.1 M NaOH. Figure 6a shows the typical i-t curve of Ni_0.7_Co_0.3_(OH)_2_/CP upon the addition of different concentrations of glucose performed at different potentials (i.e., +0.5, +0.6, and +0.7 V vs. Hg/HgO). The current increased gradually with an increasing amount of glucose at three differential voltages, demonstrating that Ni_0.7_Co_0.3_(OH)_2_ is sensitive to the change of glucose concentration. It is clear that the electrode potential at +0.6 V vs. Hg/HgO offers the best current response. The corresponding calibration graph for amperometric glucose detection on Ni_0.7_Co_0.3_(OH)_2_/CP at +0.6 V vs. Hg/HgO is shown in Figure 6b. The slope of the linear part was consistent with the change of current density that accompanied each additional mole of glucose, which indicates the sensitivity of 1541 μA•mM^−1^•cm^−2^. The detection limit was determined to be 3.42 µM (S/N = 3). The sensory performance of Ni_0.7_Co_0.3_(OH)_2_/CP is comparable to other Ni- or Co-based electrode materials (Table 1). It is worth noting that the Ni_0.7_Co_0.3_(OH)_2_/CP electrode can offer high sensitivity towards glucose detection in 0.1 M NaOH. This is attributed to the high electrochemical surface area determined by double-layer capacitance and fast electron transfer kinetics verified by EIS analysis. For comparison, amperometric glucose detection experiments were also carried out for Co(OH)_2_/CP, Ni_0.3_Co_0.7_(OH)_2_, Ni_0.5_Co_0.5_(OH)_2_, and Ni(OH)_2_ at three electrode potentials of +0.5, +0.6, and +0.7 V vs. Hg/HgO. It is clear that the sensitivities at +0.6 V vs. Hg/HgO were higher than that of +0.5 and +0.7 V vs. Hg/HgO for each sample, suggesting +0.6 V vs. Hg/HgO is the optimal electrode potential for glucose detection. Concerning the composition of the Ni-Co hydroxides, Ni_0.7_Co_0.3_(OH)_2_/CP demonstrated higher sensitivity than that of the other samples at +0.6 V vs. Hg/HgO. More detailed results of sensitivity and limit detection are shown in Table 2. 

**Figure 7:**
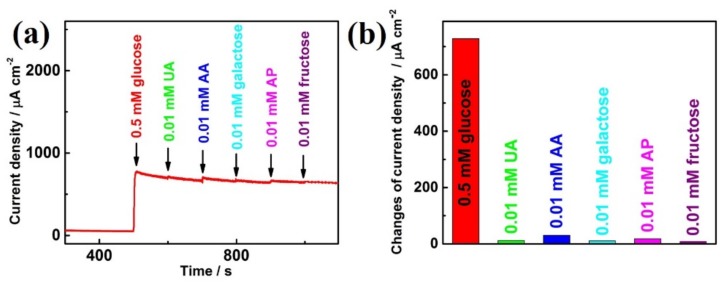
(**a**) Interference test of Ni_0.7_Co_0.3_(OH)_2_/CP with 0.5 mM glucose, 0.01 mM uric acid (UA), 0.01 mM ascorbic acid (AA), 0.01 mM galactose, 0.01 mM acetaminophen (AP), 0.01 mM fructose, and 0.5 mM glucose successively at +0.6 V in 0.1 M NaOH. (**b**) A plot that shows the changes in current density caused by the addition of glucose and other interferences.

Selectivity testing is another important factor to be considered in order to evaluate the sensory performance of Ni_0.7_Co_0.3_(OH)_2_/CP for glucose, since other bio-organic molecules, such as uric acid (UA), ascorbic acid (AA), galactose, acetaminophen (AP), and fructose, may interfere with the current response in real circumstances. Figure 7a shows the typical amperometric response of Ni_0.7_Co_0.3_(OH)_2_/CP with the addition of 0.5 mM glucose, 0.01 mM UA, 0.01 mM AA, 0.01 mM galactose, 0.01 mM AP, and 0.01 mM fructose at +0.6 V vs. the Hg/HgO electrode potential. It is clear that the addition of 0.5 mM glucose caused a significant increase in current density up to 728 μA cm^−2^, whereas the addition of a small amount of other interferences caused negligible current responses (Figure 7b). It is worth mentioning that the concentrations of interferences are much lower than that of glucose, which is reasonable since those interferences in plasma have much lower concentrations than glucose. Therefore, Ni_0.7_Co_0.3_(OH)_2_/CP can be a reasonable candidate to selectively detect glucose with a high sensitivity and low detection limits.

Accordingly, although transition metal hydroxides have been widely explored as electrode materials for nonenzymatic glucose, such as Ni(OH)_2_ [9,35,37,38,39,40], Co(OH)_2_ [10,38], and Cu(OH)_2_ [41,42], the presence of an additional transition metal indeed improved the current density response significantly, which is similar to previous works [43]. As it is shown in the XPS data, the electrode materials displayed both Ni and Co in both +2 and +3 oxidation states. Since the oxidation potentials between Ni^2+^ and Co^2+^ are similar under alkali conditions, the presence of Co^2+^ in the catalyst can enhance the oxidative current density of the Ni_0.7_Co_0.3_(OH)_2_, thus leading to enhanced sensitivity towards glucose sensing. Besides, the rational design of hollow cubic Ni_0.7_Co_0.3_(OH)_2_ nanosheets can provide a large electrochemical surface area, improved glucose diffusion pathways, and tuned conductivity that, thus, leads to an increased current density signal [44,45]. Moreover, since the electrochemical oxidation of Ni_0.7_Co_0.3_(OH)_2_ is a hydroxide ion coupled reaction pathway, the enlarged surface area and the hollow cubic structure can also boost the diffusion rate of –OH within the material. This is may be one of the reasons that this rationally designed hollow cubic structure can offer a high current density compared to other reported works. Thus, the increased oxidative current density of Ni_0.7_Co_0.3_(OH)_2_ can increase the sensitivity towards glucose sensing. 

## 4. Conclusions

Ni_x_Co_1-x_(OH)_2_ nanosheets assembled hollow cubes with controllable compositions of Ni and Co have been investigated for electrochemical glucose determination. Mixed Ni and Co metal ions in hydroxide may tune the nanostructures and conductivities of the hydroxides, thus increasing the active surface area, tuning the glucose diffusion pathways, and improving the activity of metal hydroxides towards glucose sensing. The Ni_0.7_Co_0.3_(OH)_2_/CP electrode displayed the best glucose sensory performance among other ratios, with highly selectivity and sensitivity towards electrochemical catalytic glucose oxidation. This hollow, hierarchical structure, synthetic approach could also be an effective approach for the development of other highly efficient electrode materials for nonenzymatic glucose sensors.

## Figures and Tables

**Table 1:** Summary of sensing performances of nickel, cobalt, and nanostructure-based electrodes.

Materials	Linear Range (mM)	LOD (μM)	Sensitivity (μA mM cm^−2^)	References
Ni_0.7_Co_0.3_(OH)_2_	0.002~0.8	3.42	1541	This work
AuNPS/Ni(OH)_2_	0.002~6.0	6.6	27.14	[9]
α-Ni(OH)_2_/FTO	0.01~0.75	2.5	446	[36]
Ni(OH)_2_	0.004~3.5	2.0	948	[37]
CuNps-Co(OH)_2_Nfs/Nif	0.001~0.25	0.025	43.2	[10]
CoOOH	0.003~1.109	1.37	526.8	[38]

**Table 2:** Sensitivity of Ni_x_Co_1-x_(OH)_2_/CP at different electrode potentials (vs. Hg/HgO).

Sensitivity (μA·mM^−1^·cm^−2^)	+0.5 V	+0.6 V	+0.7 V
Co(OH)_2_	518	1140	545
Ni_0.3_Co_0.7_(OH)_2_	653	1120	569
Ni_0.5_Co_0.5_(OH)_2_	301	660	646
Ni_0.7_Co_0.3_(OH)_2_	571	1541	832
Ni(OH)_2_	4.21	592	525

## Supplementary Materials

The following are available online at https://www.mdpi.com/1424-8220/19/13/2938/s1, Figure S1: The TEM images of (a) Co(OH)_2,_ (b) Ni_0.3_Co_0.7_(OH)_2_, (c) Ni_0.5_Co_0.5_(OH)_2_, and (d) Ni(OH)_2_ hollow cubes; Figure S2: Cyclic voltammograms plots of different Ni_x_Co_1-x_(OH)_2_/CP at various scan rates (10–50 mV s^−1^) scan in the non-Faradaic potential window.
