# Supplementary material for: Synthesis of Ni-Co Hydroxide Nanosheets Constructed Hollow Cubes for Electrochemical Glucose Determination
Source: Sensors (Basel). 2019 Jul 3;19(13):2938. doi: 10.3390/s19132938 (PMC6651393; doi:10.3390/s19132938)
Supplement: Supplementary file 1 [file sensors-19-02938-s001.pdf]

Supplementary Information

Synthesis of Ni-Co Hydroxide Nanosheets Constructed Hollow Cubes for Electrochemical Glucose Determination

Fengchao Sun [1,2], Shutao Wang [3], Yuqi Wang [3], Jingtong Zhang [2], Xinping Yu [2], Yan Zhou *,[1,2,3] and Jun Zhang [1,2]

1 School of Materials Science and Engineering, China University of Petroleum (East China), Qingdao 266580, China
2 School of Chemical Engineering, China University of Petroleum (East China), Qingdao 266580, China
3 College of Science, China University of Petroleum (East China), Qingdao266580, China

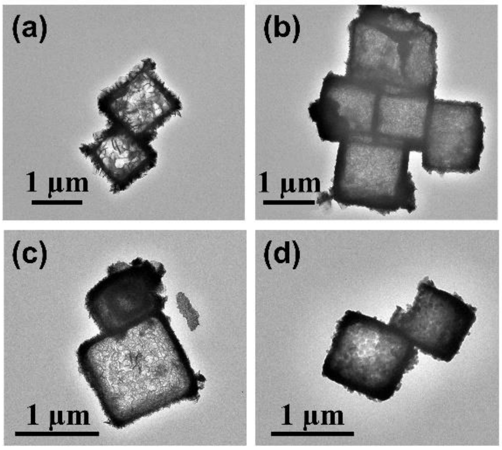

Figure S1. The TEM images of (**a**) $Co(OH)_2$, (**b**) $Ni_{0.3}Co_{0.7}(OH)_2$, (**c**) $Ni_{0.5}Co_{0.5}(OH)_2$ and (**d**) $Ni(OH)_2$ hollow cubes.

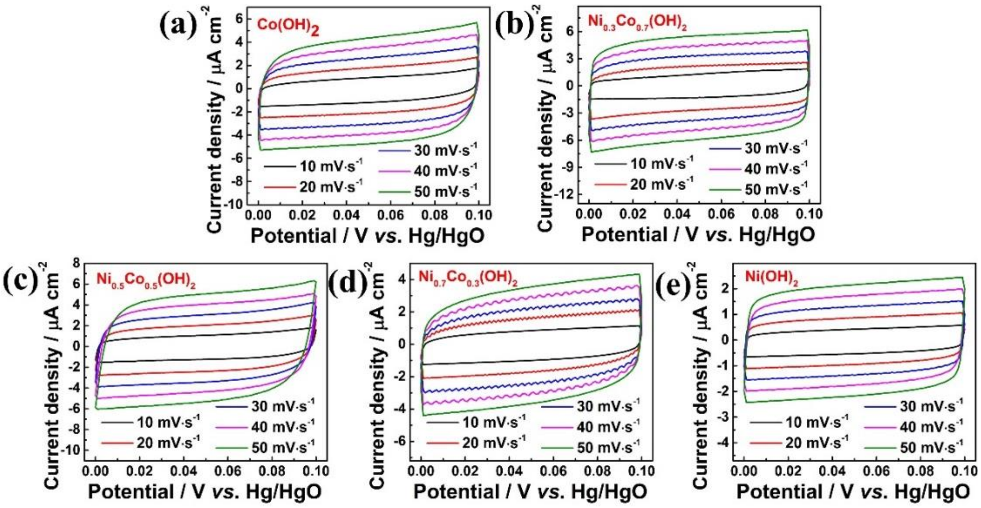

Figure S2. Cyclic voltammograms plots of different $Ni_xCo_{1-x}(OH)_2$/CP at various scan rates (10–50 mV s^{-1}).
